# Involvement of NADPH oxidase isoforms in the production of O_2^−^_ manipulated by ABA in the senescing leaves of early-senescence-leaf (*esl*) mutant rice (*Oryza sativa*)

**DOI:** 10.1371/journal.pone.0190161

**Published:** 2018-01-08

**Authors:** Zhaowei Li, Fubiao Wang, Qian Zhao, Jianchao Liu, Fangmin Cheng

**Affiliations:** 1 College of Life Sciences, Fujian Agriculture and Forestry University, Fuzhou, Fujian, China; 2 Institute of Crop Science, Zhejiang University, Hangzhou, Zhejiang, China; 3 Institute of Agricultural Ecology, Fujian Agriculture and Forestry University, Fuzhou, Fujian, China; Murdoch University, AUSTRALIA

## Abstract

In this study, the differences in reactive oxygen species (ROS) generation and abscisic acid (ABA) accumulation in senescing leaves were investigated by early-senescence-leaf (*esl*) mutant and its wild type, to clarify the relationship among ABA levels, ROS generation, and NADPH oxidase (Nox) in senescing leaves of rice (*Oryza sativa*). The temporal expression levels of *OsNox* isoforms in senescing leaves and their expression patterns in response to ABA treatment were determined through quantitative real-time reverse transcription PCR (qRT-PCR). Results showed that the flag leaf of the *esl* mutant generated more O_2^-^_ concentrations and accumulated higher ABA levels than the wild-type cultivar did in the grain-filling stage. Exogenous ABA treatment induced O_2^-^_ generation; however, it was depressed by diphenyleneiodonium chloride (DPI) pretreatment in the detached leaf segments. This finding suggested the involvement of NADPH oxidase in ABA-induced O_2^-^_ generation. The *esl* mutant exhibited significantly higher expression of *OsNox2*, *OsNox5*, *OsNox6*, and *OsNox7* in the initial of grain-filling stage, followed by sharply decrease. The transcriptional levels of *OsNox1*, *OsNox3*, and *OsFR07* in the flag leaf of the *esl* mutant were significantly lower than those in the wild-type cultivar. The expression levels of *OsNox2*, *OsNox5*, *OsNox6*, and *OsNox7* were significantly enhanced by exogenous ABA treatments. The enhanced expression levels of *OsNox2* and *OsNox6* were dependent on the duration of ABA treatment. The inducible expression levels of *OsNox5* and *OsNox7* were dependent on ABA concentrations. By contrast, exogenous ABA treatment severely repressed the transcripts of *OsNox1*, *OsNox3*, and *OsFR07* in the detached leaf segments. Therefore, *OsNox2*, *OsNox5*, *OsNox6*, and *OsNox7* were probably involved in the ABA-induced O_2^-^_ generation in the initial stage of leaf senescence. Subsequently, other oxidases activated in deteriorating cells were associated with ROS generation and accumulation in the senescing leaves of the *esl* mutant. Conversely, *OsNox1*, *OsNox3*, and *OsFR07* were not associated with ABA-induced O_2^-^_ generation during leaf senescence.

## Introduction

Leaf senescence is the final stage of leaf development and is controlled by various internal and external factors [1, 2]. This process is a genetically programmed metabolism of self-destruction as a form of programmed cell death. In this process, reactive oxygen species (ROS) act as important signaling molecules and toxic substances, which participate in the genetic regulation of leaf senescence and accelerate the completion of organ senescence [3–5].

ROS, such as superoxide radical (O_2^-^_) and hydrogen peroxide (H_2_O_2_), have been considered essential signaling molecules and key regulators of plant biological processes, including stomatal movement, pathogen defense, hormone signal transduction, programmed cell death, and plant growth and development [6–8]. Plasma membrane nicotine adenine dinucleotide phosphate (NADPH) oxidase is closely associated with the production and accumulation of ROS, which transfers electrons from cytoplasmic NADPH to O_2_ to form O_2^-^_; O_2^-^_ undergoes dismutation to produce H_2_O_2_ [9]. In the roots of wheat (*Triticum durum* D.) seedlings, NADPH oxidase participates in the nickel-induced production of ROS to respond to oxidative stress caused by nickel treatment [10]. NADPH oxidase-dependent H_2_O_2_ production is also an intermediate step in the NaCl-induced elevation of calcium (Ca) in wheat roots [11]. In *Arabidopsis*, *AtrbohD* and *AtrbohF* encoding NADPH oxidases contribute to ROS production to regulate Na^+^/K^+^ homeostasis and improve the salt tolerance of *Arabidopsis* seedlings [12, 13]. In cultured tobacco (*Nicotiana benthamiana*) cells, the accumulation of ROS induced by *NbrbohA* and *NbrbohB* is involved in resistance against pathogenic infections [14]. *NtrbohD* is necessary in ABA-induced H_2_O_2_ accumulation to improve resistance against various stresses [15]. In maize (*Zea mays*), *ZmrbohA*, *ZmrbohB*, *ZmrbohC* and *ZmrbohD* are responsible for the biphasic response of ROS generation in ABA signaling transduction [16]. Therefore, NADPH oxidase is the main source of ROS in plant tissues and suspension culture cells; this enzyme plays important roles in regulating biological processes and responding to diverse environmental stimuli and aging factors [13, 17, 18]. In rice, nine genes encoding NADPH oxidase have been identified within the genome, and individual *OsNox* isoforms exhibit unique stress-response characteristics and distinct functions in response to various environmental stresses [19, 20]. *OsNox2* (*OsrbohA*) and *OsNox6* (*OsrbohE*) participate in the regulations of ROS-dependent signaling pathways in plant immune response [21]. Therefore, NADPH oxidase-induced ROS production is involved in multiple signaling pathways to respond to various stresses and aging factors; individual isoforms of NADPH oxidase exhibit distinct regulation patterns to manipulate ROS generation [20, 22]. However, the accurate molecular functions of *OsNox* isoforms manipulating ROS generation during the leaf senescence of rice remain ambiguous and thus should be investigated.

The function of NADPH oxidase in ROS generation in response to various stresses in plant tissues is mediated by abscisic acid (ABA). ABA plays important regulatory roles in plant responses and adaptation to various stressors, including drought, salinity, low temperature, and other biotic and abiotic factors [23, 24]. NADPH oxidase is involved in ABA-induced ROS production; as a result, antioxidant defense systems against oxidative damage are also stimulated to respond to various stress conditions [17, 25, 26]. NADPH oxidase-induced ROS generation involves rate-limiting second messengers in ABA signaling [27]. ABA-induced ROS production via NADPH oxidase is involved in the closure of stomata and the activation of plasma membrane Ca^2+^ channels in leaf guard cells; thus, responses to environmental stresses are stimulated [15, 28]. During leaf senescence, ROS accumulate and antioxidant enzymes change in senescing leaves; NADPH oxidase activities and ABA levels are also enhanced in senescing leaves [29, 30]. H_2_O_2_ and ABA are key regulatory factors that mediate the progression of leaf senescence; exogenous H_2_O_2_ or ABA application can induce or accelerate leaf senescence [5, 29–31]. However, studies have yet to fully elucidate the regulatory mechanism of NADPH oxidase involved in ROS generation and ABA signaling during leaf senescence. The molecular patterns of NOX isoforms implicated in ROS generation in response to ABA should be further investigated in the senescing leaves of rice.

In this study, genotypic differences in ROS generation and ABA accumulation were investigated in the leaves of two rice cultivars, namely, early-senescence-leaf (*esl*) mutant and its corresponding wild type. The enhancing effects of exogenous ABA-induced O_2^-^_ production from NADPH oxidase were analyzed in detached leaf segments. The temporal expression levels of *OsNox* isoforms during leaf senescence and their expression patterns in response to ABA treatment were determined through quantitative real-time reverse transcription PCR (qRT-PCR) to clarify the possible relationship among *OsNox* isoform transcription, O_2^-^_ generation, and ABA levels in senescing leaves.

## Materials and methods

### Plant materials and experimental treatments

Two rice cultivars, namely, an *indica* rice cultviar (Fu142) and its mutant with an *esl* phenotype, were used in this study. The *esl* mutant was derived from Fu142 cultivar (*Oryza sativa* L. ssp. *indica*) by gamma-irradiated mature seeds, and the stable *esl* inherited mutant was obtained by successive self-pollination. The identification of plant phenotype was performed from the M2 to M8 generations. M9 seeds were used in this experiment. The *esl* mutant was similar to the wild-type cultivar in plant morphology, plant height, and growth period until the late tillering stage. No visible differences were observed between the *esl* mutant and the wild-type cultivar in seedling and early tillering stages. However, the flag leaf of the *esl* mutant displayed exacerbated lesions and accelerated senescence symptoms in the grain-filling stage. The rusty lesions initially appeared on the tip of the leaf blade and then progressively spread downward to cover the whole leaf surface. Finally, the whole flag leaf of the *esl* mutant was nearly withered at approximately the 20th day of the grain-filling stage; as a result, agronomic traits and grain yield deteriorate [5].

Rice seeds were sown in a seedling nursery on April 25 and transplanted on May 20. Field experiments were performed at the experimental farmland of Zijingang campus (30°18´N, 120°04´N), Zhejiang University in Hangzhou, China. The field plots were arranged by following a random design with three replications for each cultivar. Each replication was planted in 10 × 12 rows, and plant spacing was 18 cm × 18 cm, with one rice seedling for each hill. The field trail was managed in accordance with local cultivation practices. Soil type was periodically waterlogged paddy soil, with 1.69 g/kg total N, 24.5 mg/kg available P, and 103.7 mg/kg exchangeable K. The rice plants were sampled in the grain-filling stage. A total of 50–70 rice plants with uniform anthesis time were randomly selected and tagged in each plot, and the flag leaves of the tagged plants were sampled during a 7-day interval, with three independent biological replicates at 9:00 a.m. The fresh samples were immediately frozen in liquid nitrogen and kept at −80°C for further experimental analyses.

Two supplement experiments were conducted using the detached leaf segments to investigate the effect of exogenous ABA on *OsNox* isoforms with respect to O_2^-^_ generation. The fully extended leaves on the topmost position of rice plants were carefully detached in the booting stage. The leaves from rice plants at that time remained green and did not exhibit visual stress symptoms. In Expt. I (incubating concentration treatment), the detached leaf segments of rice plant were exposed to four ABA concentration treatments: 10, 50, 100, and 500 μM [5]. For each treatment, 25 mL of ABA solution was added to Petri dishes, with 4 dishes for each incubating concentration. After 6 h of incubation at 28°C in darkness, the leaf segments were sampled for subsequent analysis. In Expt. II (incubating duration treatment), the topmost fully extended leaves of rice plants were detached in the booting stage, and the detached leaf segments were floated on the solutions containing 25 mL of 100 μM ABA in Petri dishes placed at 28°C in darkness [5]. The leaf segments were subsequently sampled at 0, 0.5, 1, 3, 6, and 12 h after incubation. Before these immersions were performed, the leaf segments were placed in distilled water for 2 h to eliminate wound stress. The samples exposed to distilled water were as control, and three replications were prepared for Expt. I and II.

### Determination of leaf O_2^-^_ production

The production of O_2^-^_ in sample was measured through Wang’s method with a slight modification [32]. Fresh leaf sample (0.50 g) was homogenized with 5 mL of 65 mM potassium phosphate buffer (pH 7.8) in ice, and then the homogenate was centrifuged at 10000 g for 15 min at 4°C, and 2 mL of supernatant was mixed with 0.4 mL of 10 mM hydroxylamine hydrochloride and incubated for 20 min at 25°C to produce NO_2^-^_, and then 2 mL of 17 mM sulphanilic acid and α-naphthylamine were added separately, followed by incubating for 20 min at 25°C. Subsequently, 6 mL of trichloromethane was added into the mixture and shook well, and centrifuged at 10 000 g for 3 min. The upper pink aqueous phase was measured at 530 nm by a spectrophotometer. The production rate of O_2^-^_ was calculated according to the standard NaNO_2_ concentration gradient using the same procedures.

### Tissue localization of O_2^-^_

The localization of O_2^-^_ was implemented as Romero-Puertas’s protocol with a slight modification [33]. Superoxide in leaf reacts with nitroblue tetrazolium chloride (NBT) and produces the blue formazan precipitates. Leaf segments were gently immersed in a 0.1% solution of NBT in 50 mM potassium phosphate buffer (pH 6.4), containing 10 mM Na-azide and 0.01% tween-20, and then were illuminated until appearance of dark spots, characteristic of blue formazan precipitates. After that, the leaf segments were bleached by immersing in boiling ethanol for 20 min until spots were clearly visible.

### ABA analysis

Endogenous ABA analysis was carried out using the UPLC-ESI-qMS/MS method [34]. Fresh leaf samples were crushed to a fine powder, and then soaked in 1 mL of extraction solvent (methanol: formic acid: water = 15: 1: 4) at −30°C for 24 h. After centrifugation at 10000 g for 15 min, the supernatant was transferred to a 96-well collection plate, and the pellet was re-extracted with 0.2 mL of extraction solvent, before combining with the first supernatant. The supernatant was evaporated and then reconstituted with formic acid, and subjected to UPLC-ESI-qMS/MS analysis [34].

### RNA isolation and cDNA preparation

Total RNA was extracted from the leaf samples with Trizol reagent according to the manufacturer’s protocol (Invitrogen, Carlsbad, CA, USA). The RNA quality was checked with a spectrophotometer (NanoDropTM 1000, Thermo Fisher Scientific, USA), and then 1 μg of RNA was treated with 1 unit of DNaseI (Promega) at 37°C for 15 min to remove the possible contamination of genomic DNAs. The ReverTra Ace qPCR reverse transcriptase Kit (TOYOBO, OSAKA, JAPAN) was used for cDNA synthesis with an oligo (dT) primer. The reaction was conducted at 37°C for 15 min and then stopped by heating at 95°C for 5 min. The concentration of cDNA was about 20 ng μL^-1^.

### Real-time fluorescence quantitative PCR

Aliquots of cDNA mixtures were used as the templates for quantitative real-time PCR analysis by SYBR Green Real-time PCR Master Mix reagent Kit (TOYOBO, OSAKA, JAPAN). Reactions were performed on a Bio-Rad CFX96 (Bio-Rad, USA) according to the manufacture’s protocols. 1 μL cDNA was added to 10 μL SYBR buffer, and 1.6 μL 10 mM primer pairs in a final 20 μL reaction volume. The gene-specific primer pairs were designed by Primer Premier 5.0 (Premier, Canada) as listed in Table 1. The qRT-PCR conditions were 94°C for 30 s, followed by 40 cycles of 94°C for 5 s, 58°C for 10 s, and 72°C for 15 s. To verify the specificity of each primer set and optimize PCR conditions of the annealing temperature and PCR efficiency, the fluorescence signal specificity of PCR amplification was detected for each primer pairs and their melting curve (from 58°C to 94°C) was examined prior to the experimental measurements. *Actin* was used as the internal control gene. The samples were normalized using *Actin* expression, and the relative expression levels were analysed using the 2^-ΔΔCT^ method [35]. The average value and standard error were calculated from three independent biological replicates.

**Table 1:** Sequence of primers for *ACTIN* and *OsNox* isoform genes used for real-time quantitative polymerase chain reaction.

Gene	Accession No.	Primer pairs		Products (bp)
		Forward primer (5’ → 3’)	Reverse primer (5’ → 3’)	
*ACTIN*	X16280	5’-CAGCACATTCCAGCAGATGT-3’	5’-TAGGCCGGTTGAAAACTTTG-3’	198
*OsNox1*	NM_001049555	5’-GCGATGCTCGTTCTGCTCTC-3’	5’-GGTCGTGCGAAATGGGTCTT-3’	106
*OsNox2*	NM_001050700	5’-CAAGCCAAGCACTGAGCCA-3’	5’-GAACAGTCCAGCCATTATCCC-3’	149
*OsNox3*	NM_001051260	5’-GGCATCCCTTCTCCATCAC-3’	5’-CTCGCAAGCCTTCCCAAAC-3’	113
*OsNox4*	NM_001062318	5’-CGCTACTCCGTGGTATGGTGA-3’	5’-GTCTTGTTGAAACGCCTCTGC-3’	127
*OsNox5*	NM_001062650	5’-TTGGGATAATGGCTGGGTTG-3’	5’-TGGTGTTGCGGCATACTGG-3’	159
*OsNox6*	NM_001068491	5’-CTCCTCATCGTCGTCTACCTCC-3’	5’-AAAGCATCAATGGCACAGCA-3’	112
*OsNox7*	NM_001069802	5’-CCGAACAAACGGAGACTGGA-3’	5’-CGCCTAGCTCGCTGAATGAA-3’	101
*OsFR01*	NM_001060176	5’-CACCACCTCTACGCCCTCTT-3’	5’-GAACACGCCAGGCAGGAT-3’	84
*OsFR07*	NM_001059431	5’-TGCGGAACCGTGGAACTA-3’	5’-CTCCCTCACCTGAACGAAGA-3’	84

### Statistical analysis

All determinations were performed in at least three independent experiments. Statistical differences were analyzed by analysis of variance (ANOVA) using the SPSS statistical software package (Chicago, USA). Differences were considered significant at a probability level of P ≤ 0.05 or 0.01. Standard deviation (SD) was calculated and shown in the figures.

## Results

### ABA enhanced the production of O_2^-^_ during leaf senescence

**Fig 1:**
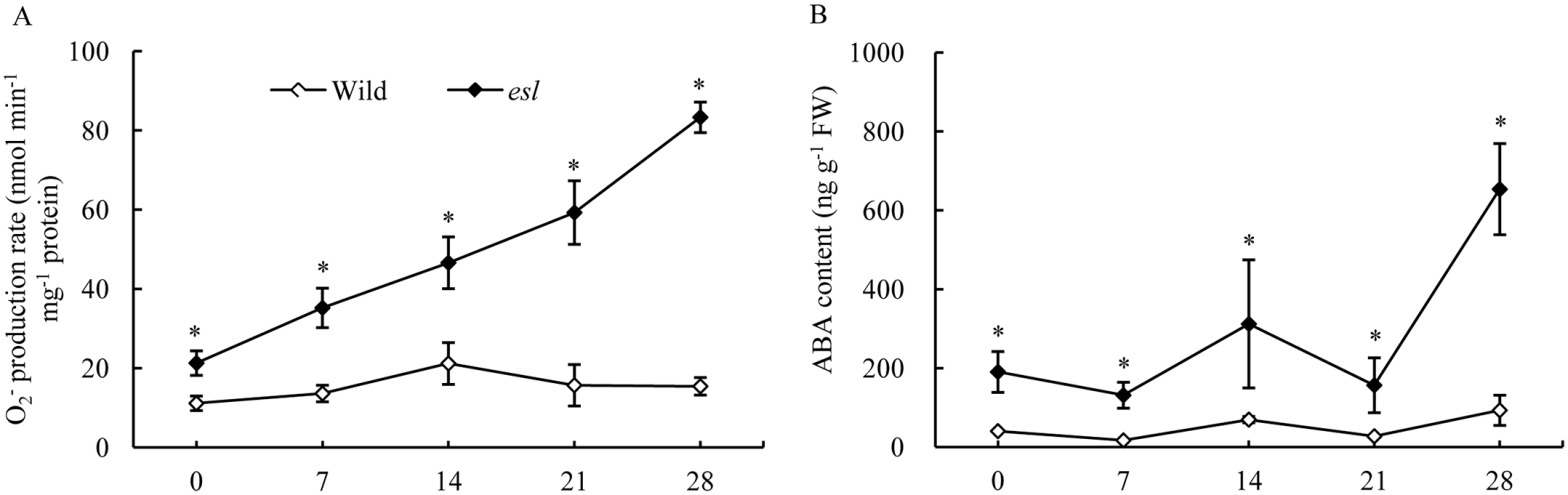
O_2^-^_ production rate and ABA contents in the flag leaves of two rice cultivars in the grain-filling stage. Vertical bars represent standard errors (n = 3).

As reported in our previous study, the flag leaf of the *esl* mutant rice exhibited early senescence symptoms and significantly higher H_2_O_2_ level than the wild-type cultivar did in the grain-filling stage, and increasing H_2_O_2_ was closely associated with the regulation of leaf senescence in *esl* mutant rice after anthesis [5]. In this study, the flag leaf of the *esl* mutant showed significantly higher and progressively enhancive O_2^-^_ production rate than its wild-type cultivar did in the grain-filling stage (p ≤ 0.05) (Fig 1A, S1 Table). The endogenous ABA level in senescing flag leaf of *esl* mutant was significantly higher than that in wild-type cultivar (p ≤ 0.05) (Fig 1B, S1 Table), suggesting that the production of O_2^-^_ in the senescing flag leaf of the *esl* mutant was probably associated with ABA level in the grain-filling stage.

**Fig 2:**
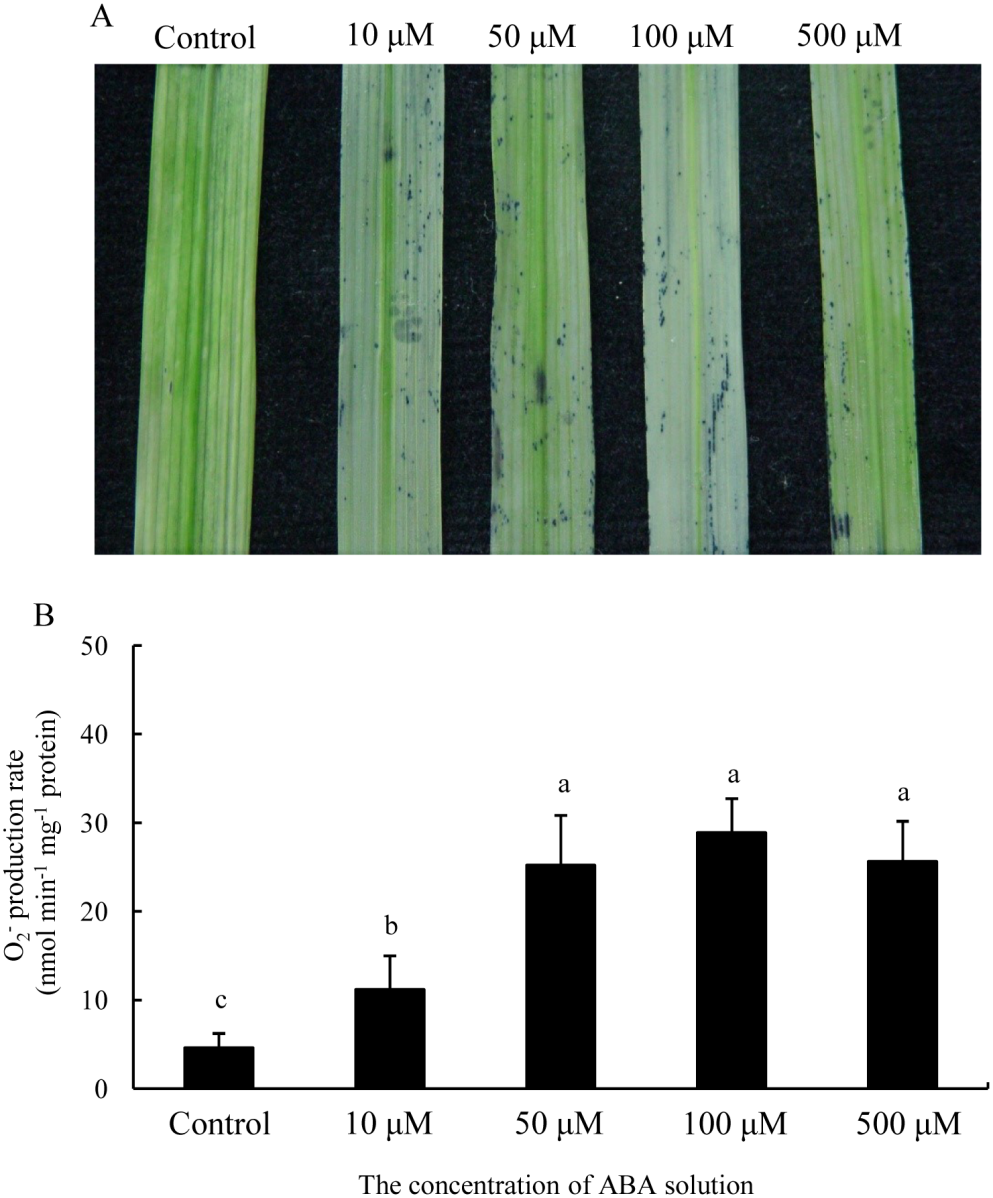
O_2^-^_ production in detached leaf segments incubated by various concentrations of exogenous ABA solutions. Significant differences (p ≤ 0.05) between ABA doses are indicated by different letters.

The detached leaf segments were incubated in exogenous ABA solutions with different concentrations to demonstrate the effect of ABA on O_2^-^_ generation in the senescing leaf. The tissue localization of O_2^-^_ showed that exogenous ABA treatments obviously enhanced the formation of blue formazan deposit in the detached leaf segments (Fig 2A), and the production rate of O_2^-^_ in detached leaf segments was significantly enhanced by various concentrations of ABA solutions (p ≤ 0.05) (Fig 2B, S2 Table).

**Fig 3:**
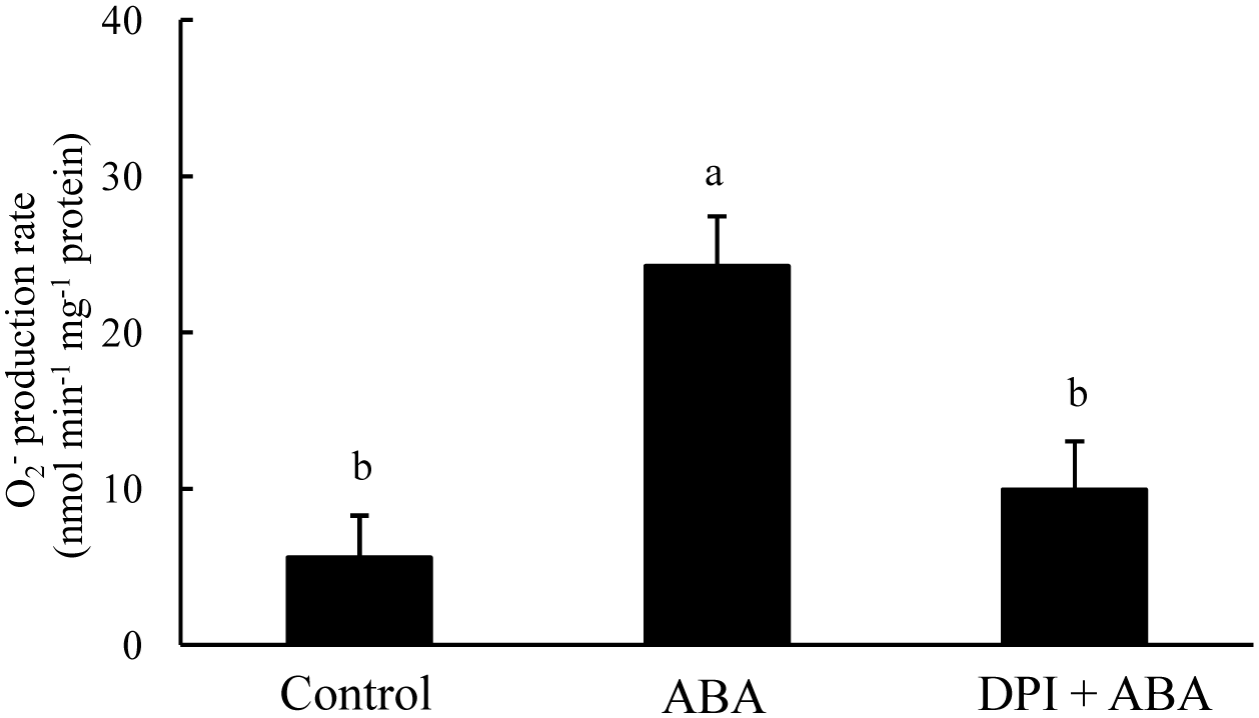
Involvement of NADPH oxidase in ABA-induced O_2^-^_ production in detached leaf segments. **The DPI concentration is 25**μM, and ABA concentration is 100μM; **Significant differences (p ≤ 0.05) between different treatments are indicated by different letters**.

Diphenyleneiodonium chloride (DPI), an inhibitor of NADPH oxidase, was used to investigate the involvement of NADPH oxidase in ABA-induced O_2^-^_ production. The detached leaf segments were firstly incubated in DPI solution for 6 h and then immersed in exogenous ABA solution. In Fig 3, O_2^-^_ generation caused by exogenous ABA in the detached leaf segments was repressed by DPI pretreatment (p ≤ 0.05, S3 Table); thus, ABA-induced O_2^-^_ production was primarily associated with NADPH oxidase.

### Genotypic-dependent differences in the transcriptional profile of various *OsNox* isoform genes during leaf senescence

**Fig 4:**
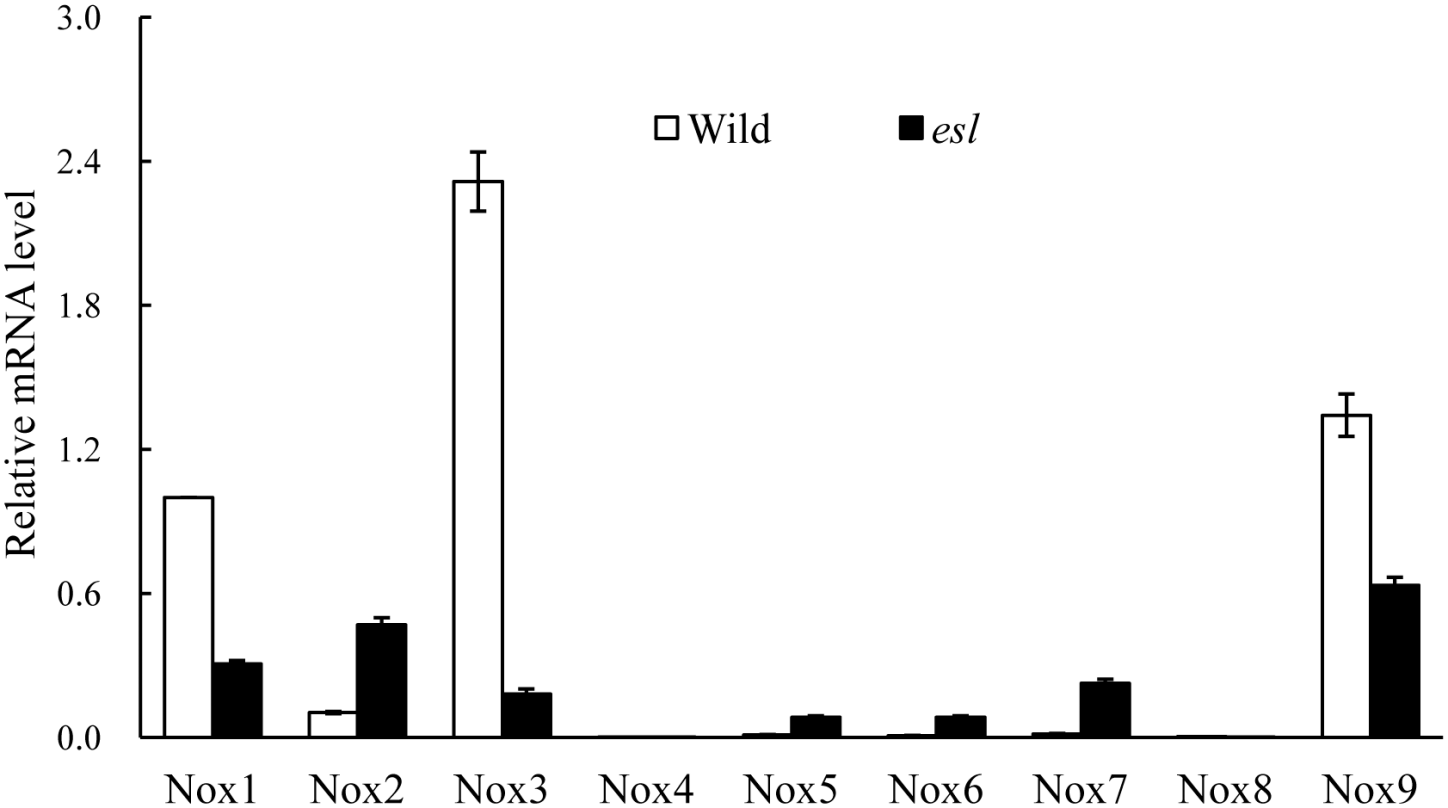
Comparison of the expression of *OsNox* isoforms in the flag leaves of two rice cultivars in the booting stage.

The *esl* mutant displayed significantly higher transcripts for *OsNox2*, *OsNox5*, *OsNox6*, and *OsNox7* isoforms in the flag leaf than the wild-type cultivar did. The *esl* mutant exhibited strikingly lower expression abundance for *OsNox1*, *OsNox3*, and *OsFR07* isoforms in the heading stage than the wild-type cultivar did (Fig 4, S4 Table). The transcripts of *OsNox4* and *OsFR01* were not detected in the leaves of the two rice cultivars (Fig 4).

**Fig 5:**
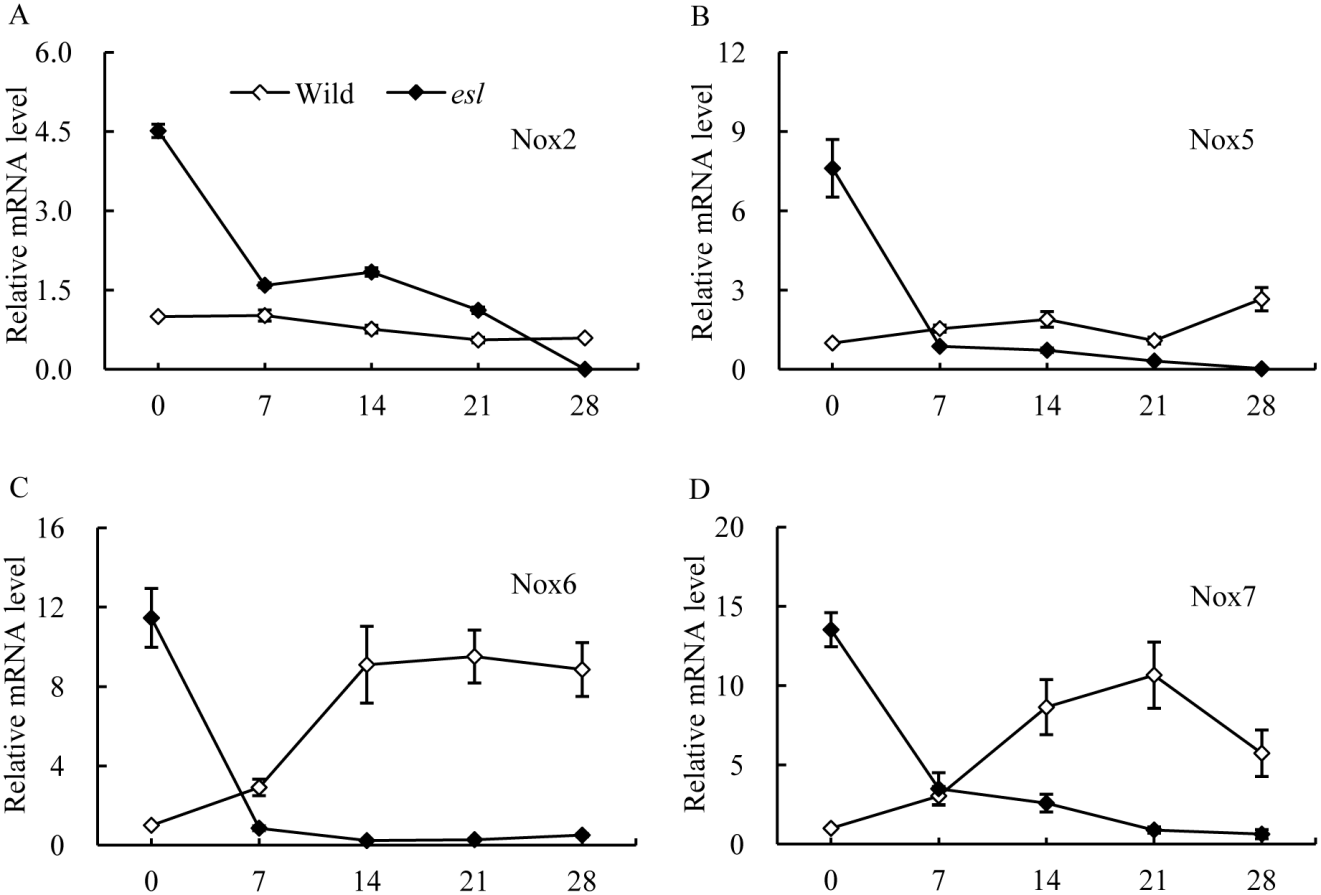
Temporal expression patterns of *OsNox2*, *OsNox5*, *OsNox6*, and *OsNox7* in the flag leaves of two rice cultivars in the grain-filling stage.

**Fig 6:**
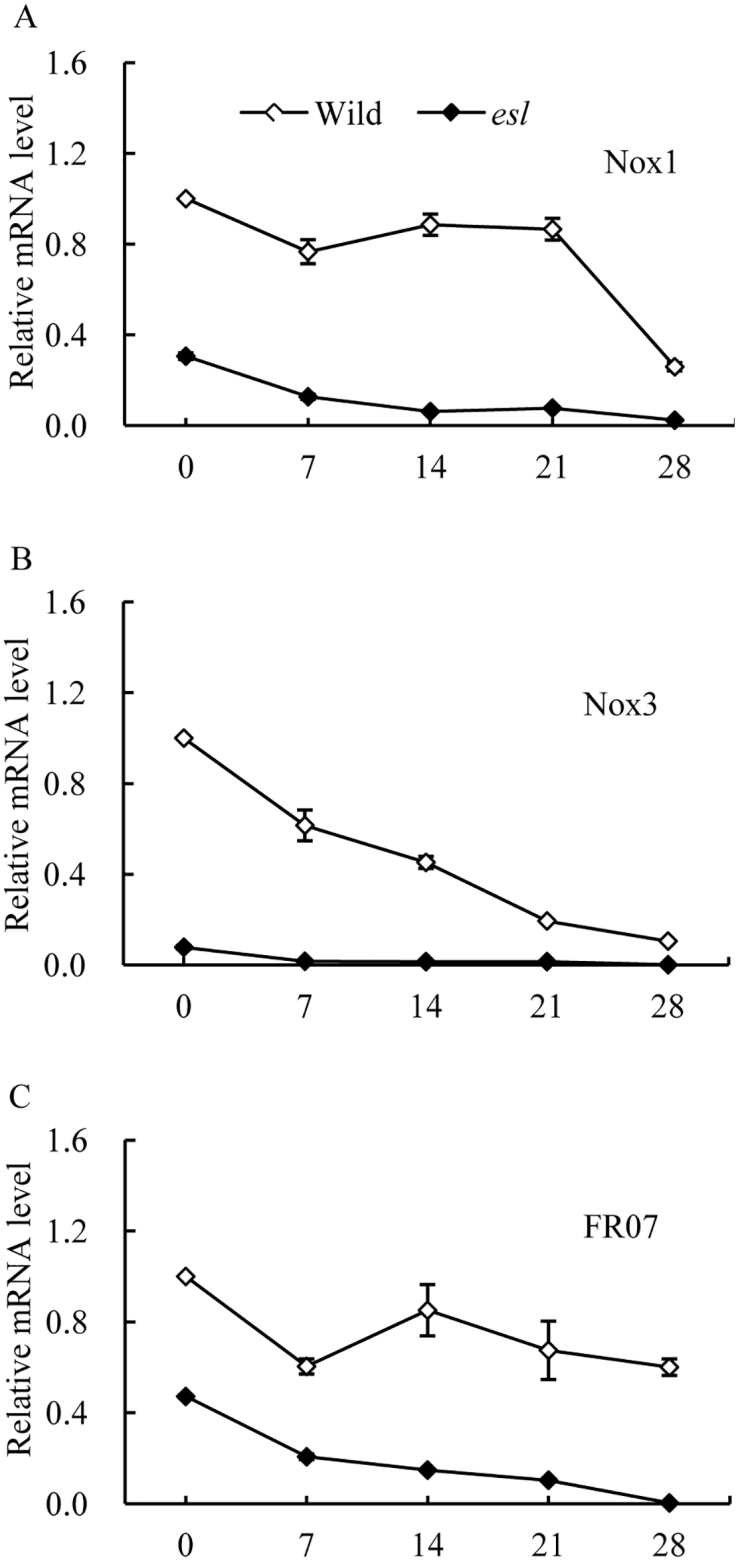
Temporal expression patterns of *OsNox1*, *OsNox3*, and *OsFR07* in the flag leaves of two rice cultivars in the grain-filling stage.

In Fig 5, the temporal transcriptional patterns of *OsNox2*, *OsNox5*, *OsNox6*, and *OsNox7* in the flag leaf of the *esl* mutant were strikingly higher than those in the wild-type cultivar in the initial stage of grain filling, and then sharply decreased. By contrast, the wild-type cultivar exhibited relatively consistent expression patterns of *OsNox2* and *OsNox5* in the grain-filling stage, and increasing expression levels of *OsNox6* and *OsNox7* in the mid-late stage of grain filling (S5 Table). However, the temporal expression patterns of *OsNox1*, *OsNox3* and *OsFR07* isoforms in the flag leaf of *esl* mutant were significantly lower than those in wild type during the whole grain-filling stage (Fig 6, S5 Table). These results suggested that the transcripts of *OsNox2*, *OsNox5*, *OsNox6*, and *OsNox7* isoforms were probably associated with the O_2^-^_ generation in the senescing flag leaf of the *esl* mutant in the initial stage of grain filling.

### Association of ABA-induced O_2^-^_ generation with the transcripts of various *OsNox* isoforms in the detached leaf segments

**Fig 7:**
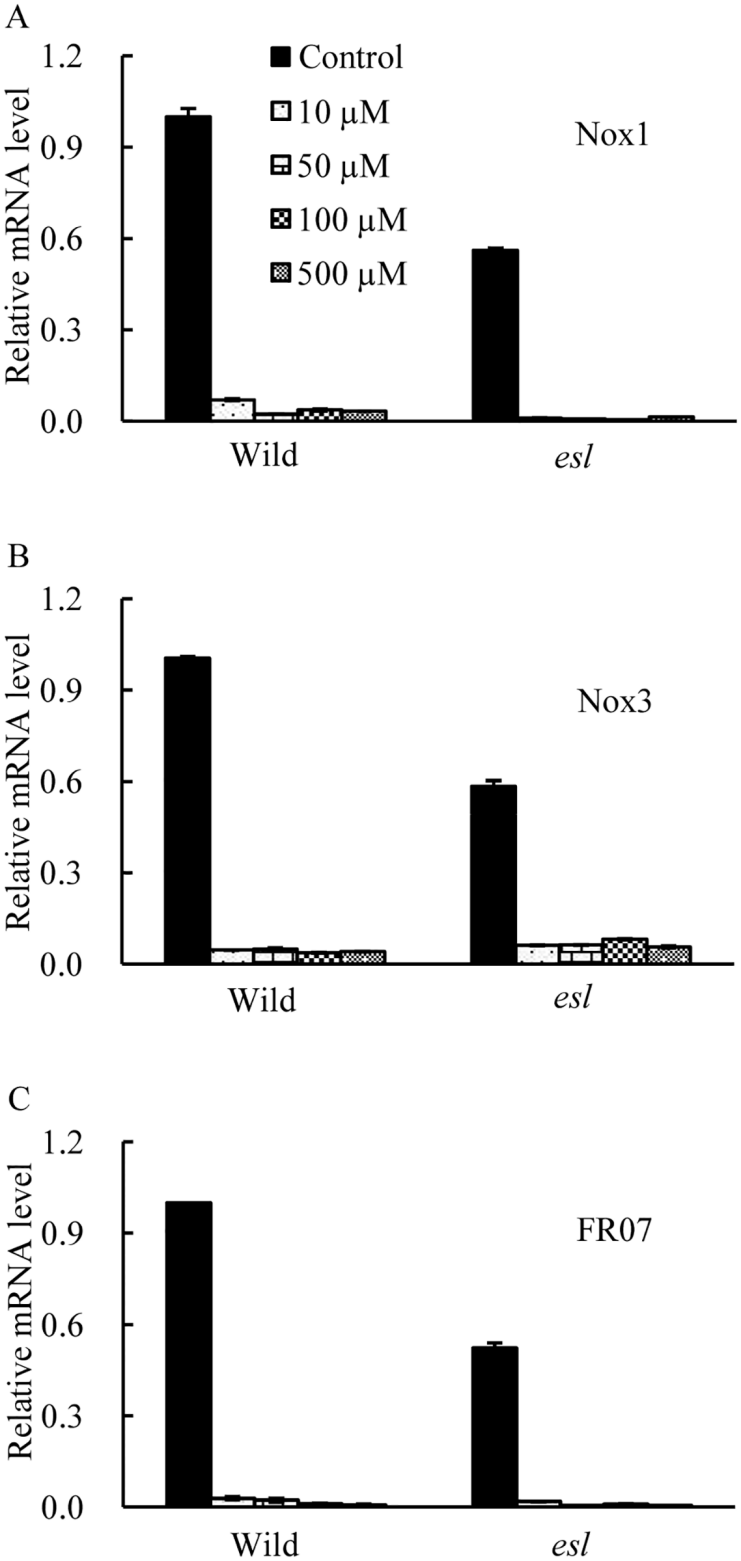
Transcriptional analyses of *OsNox1*, *OsNox3*, and *OsFR07* in the detached leaf segments of two rice cultivars incubated at 10, 50, 100, and 500 μM ABA solutions for 6 h.

**Fig 8:**
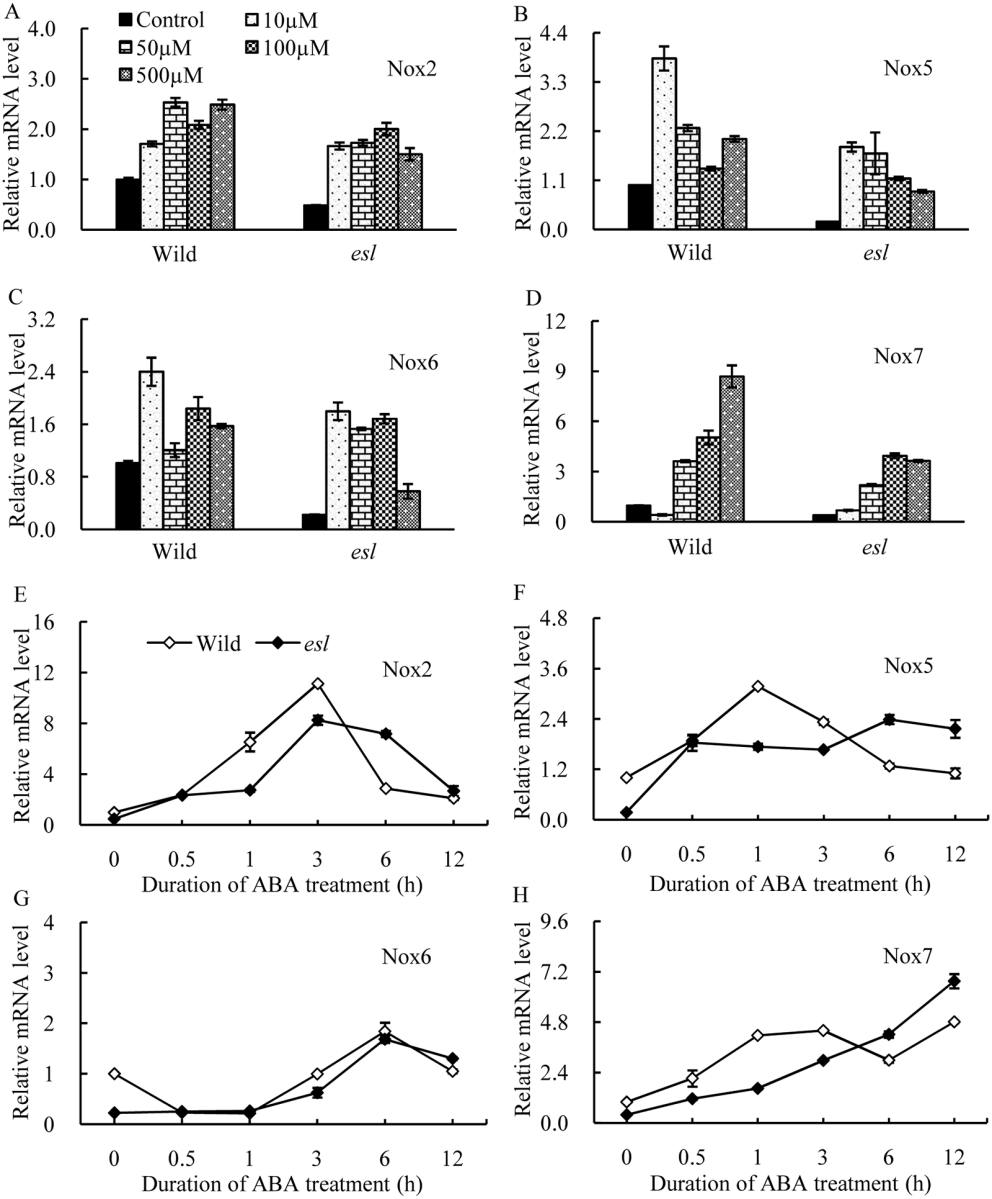
Transcriptional analyses of *OsNox2*, *OsNox5*, *OsNox6*, and *OsNox7* in the detached leaf segments of two rice cultivars treated with exogenous ABA solutions. A, B, C, and D respectively indicate the comparison of the expression levels of *OsNox2*, *OsNox5*, *OsNox6*, and *OsNox7* treated with various concentrations of exogenous ABA solutions for 6 h; E, F, G, and H respectively illustrate the temporal expression patterns of *OsNox2*, *OsNox5*, *OsNox6*, and *OsNox7* treated with 100 μM exogenous ABA solution.

The transcriptional expression levels of *OsNox* isoforms in response to exogenous ABA were investigated in the detached rice leaf segments to clarify the regulatory relationship between ABA and *OsNox* transcripts involved in O_2^-^_ generation. In Fig 7, exogenous ABA treatments severely repressed the transcripts of *OsNox1*, *OsNox3*, and *OsFR07* regardless of ABA concentrations (S6 Table), suggesting that these three *OsNox* isoforms were negatively correlated with ABA hormone and were not involved in the ABA-induced O_2^-^_ generation. By contrast, the transcripts of *OsNox2*, *OsNox5*, *OsNox6*, and *OsNox7* were significantly enhanced by exogenous ABA treatments (Fig 8A–8D, S6 Table). The enhanced expression levels of *OsNox2* and *OsNox6* caused by ABA treatment were variable with the duration of ABA treatment, regardless of rice genotypes or ABA concentration (Fig 8A and 8C). The transcript of *OsNox2* reached its peak level at 3 h after 100 μM ABA treatment. *OsNox6* reached the highest expression at 6 h after ABA treatment (Fig 8E and 8G). The effects of exogenous ABA on the transcripts of *OsNox5* and *OsNox7* in the two rice cultivars were dependent on ABA concentrations. For instance, *OsNox5* displayed the highest expression at 10 μM ABA; by contrast, the expression of the corresponding transcript was reduced in two rice cultivars as the ABA concentration was increased (Fig 8B). The temporal expression pattern of *OsNox5* held relatively consistent after 0.5 h incubated in 100 μM ABA solution (Fig 8F). *OsNox7* showed a promoting transcript pattern with the increase in ABA concentration (Fig 8D). The temporal expression levels of *OsNox7* in the two rice cultivars were gradually enhanced with the duration of ABA treatment and exhibited a dependence on the duration of ABA treatment (Fig 8H). These results indicated that the response expression of *OsNox*5 and *OsNox7* to exogenous ABA treatment varied widely depending on ABA levels. *OsNox5* responded to low ABA level; conversely, *OsNox7* was mainly involved in the response to high ABA level. Such diversity possibly played a complementary role in detecting the changes in ABA and in inducing O_2^-^_ production at various ABA levels during leaf senescence.

## Discussion

ROS as ubiquitous messengers of stress responses likely play a signaling role in various adaptive processes [36]. ROS production by NADPH oxidase is involved in the ABA signaling pathway to activate appropriate responses and acclimate under various stress conditions [4, 10, 17]. Under water stress condition, ROS originated from NADPH oxidase participates in ABA signal transduction; as a result, antioxidant enzyme activity is enhanced [37]. ROS, such as H_2_O_2_ derived from NADPH oxidase, has been considered a key factor mediating programmed cell death and tissue aging in plants [36, 38]. In most species, the distinctive feature of plant senescence is the increase in the levels of ROS and ABA hormone, accompanied by changes in enzyme activities related to ROS production and scavenging [5, 39]. H_2_O_2_ is involved in the ABA-induced leaf senescence in rice [29]. Our previous study demonstrated that H_2_O_2_ is required for leaf senescence, and ABA-induced H_2_O_2_ generation is closely associated with the regulatory metabolism of leaf senescence [5]. In the present study, the flag leaf of the *esl* mutant promoted the O_2^-^_ production and increased the ABA levels during leaf senescence (Fig 1, S1 Table). The exogenous ABA treatment induced the O_2^-^_ production in the detached leaf segments (Fig 2, S2 Table). The DPI pretreatment severely repressed the ABA-induced O_2^-^_ production in the detached leaf segments (Fig 3, S3 Table). Therefore, the present results implied that the ABA-induced O_2^-^_ production was probably involved in the regulation of leaf senescence process. NADPH oxidase was required for the O_2^-^_ production during ABA signal transduction. Thus, NADPH oxidase is closely associated with the ROS-dependent regulation of ABA-induced leaf senescence.

Plant NADPH oxidase has been known as a respiratory burst oxidase homolog (rboh) and is homologous to the catalytic subunit (gp91phox) of a phagocytic NADPH oxidase [40]. In *Arabidopsis*, at least 10 *rboh* isoforms are found, *AtrbohB* plays an essential regulatory role in the embryogenesis of germinating seeds, and *AtrbohC* is likely involved in root hair development [40–42]. *AtrbohD* and *AtrbohF* exhibit the multifarious functions in pathogen recognition and stomatal regulation [12, 27]; these findings indicate that distinct *rboh* isoforms in *Arabidopsis* show functional diversity in plant development and stress responses. In rice, nine *OsNox* isoforms possibly perform diverse functions in stress response and tissue development [21]. The NOX activation is mediated by cytosolic Ca^2+^ spike during stress response [22]. Exogenous Ca^2+^ treatment increases the NADPH oxidase activity in leaves [37]. In our study, the flag leaf of the *esl* mutant showed significantly lower transcript levels for *OsNox1*, *OsNox3*, and *OsFR07* than the flag leaf of the wild-type cultivar did (Figs 4 and 6, S4 Table); this result was probably attributed to low Ca^2+^ levels in the senescing leaf of the *esl* mutant (data not shown). Furthermore, the deprivation of apoplastic Ca^2+^ by EGTA chelation significantly depressed the transcripts of *OsNox1*, *OsNox3*, and *OsFR07* in the detached leaf segment. In contrast, the expression of *OsNox1* and *OsNox3* is strongly stimulated by Ca^2+^ or drought treatments; the expression of *OsFR07* is upregulated by salt stress [20]. In *Arabidopsis rhd2* mutant, that lacks a functional *AtrbohC* gene and exhibits a defective root hair growth, *AtrbohC* controls the development of root hair by producing ROS that regulated plant cell expansion through the activation of Ca^2+^ channels [18]. Besides, *AtrbohC* (At5g51060) of *Arabidopsis thaliana* possesses the close relationship or similar functions with *OsNox1* of *Oryza sativa* [20]. Therefore, *OsNox1*, *OsNox3*, and *OsFR07* were likely associated with Ca^2+^ signal in rice, and the low Ca^2+^ content in senescing leaf limited the transcripts of *OsNox1*, *OsNox3*, and *OsFR07* in the *esl* mutant. Exogenous ABA treatment repressed the transcripts of *OsNox1*, *OsNox3*, and *OsFR07* in the detached leaf segments (Fig 7, S6 Table). Therefore, these isoforms were probably not the major participators which involved in ABA signaling during leaf senescence.

The ROS production induced by the expression of *OsNox2* is involved in Ca^2+^ signaling and in response to plant immune stress [21]. The ROS generation stimulated by *OsNox2* is closely associated with the regulation of plant development and drought response [20]. The expression of *OsNox5* is upregulated in the leaves of rice under drought or high temperature conditions [20]. A study on *OsrbohA* and *OsrbohE* knockdown rice plants revealed that *OsrbohA*(*OsNox2*) and *OsrbohE* (*OsNox6*) are involved in the ROS production in suspension culture cells of rice; after these cells are inoculated with pathogens at different intervals, *OsNox2* contributes to ROS production in the early phase, whereas *OsNox6* is responsible for ROS production in the late phase; thus, signaling pathways are regulated at different phases during immune responses [21]. In our study, the flag leaf of the *esl* mutant yielded higher expression levels of *OsNox2*, *OsNox5*, *OsNox6*, and *OsNox7* than the flag leaf of the wild-type cultivar did in the initial stage of leaf senescence; the expression levels then decreased sharply until day 7 post-anthesis (Figs 4 and 5). Studies on *dnd1* mutant plants with mutation in the gene encoding the plasma membrane-localized Ca^2+^-conducting CNGC2 channel, have revealed that the appearances of early senescence-associated phenotypes are accompanied by decreased Ca^2+^ levels in *dnd1* leaves; the application of a Ca^2+^ channel blocker hastens the senescence of detached wild-type leaves [43]. Therefore, our results indicated that *OsNox2*, *OsNox5*, *OsNox6*, and *OsNox7* in the *esl* mutant were probably responsible for ROS production in the initial stage of leaf senescence; afterward, the remarkable decrease in the transcripts of *OsNox2*, *OsNox5*, *OsNox6*, and *OsNox7* in the *esl* mutant was likely regulated by decreasing Ca^2+^ levels in senescing leaves.

Recently, a novel rice C2H2-type zinc finger protein, ZFP36, has been discovered, which was involved in ABA-induced antioxidant defense by regulating the expression of *OsrbohE* (*OsNox6*) and *OsrbohB* (*OsNox7*), suggesting *OsNox6* and *OsNox7* were essential for ABA signaling [44]. Besides, ABA treatment stimulated NOX activity to produce ROS in plant guard cells in response to ABA [17, 45], whereas the transcripts of Nox isoform genes were affected by exogenous or endogenous ABA level [15, 27]. In this study, the expression levels of *OsNox2*, *OsNox5*, *OsNox6*, and *OsNox7* were significantly enhanced by exogenous ABA treatment (Fig 8A–8D). Among them, the expressions of *OsNox5* and *OsNox7* were dependent on ABA concentrations (Fig 8B and 8D). Thus, *OsNox5* and *OsNox7* were associated with distinct ABA concentrations in plant tissues. *OsNox5* responded to low ABA levels, and *OsNox7* was probably involved in the response to high ABA levels, thereby possibly playing a complementary role in detecting changes in ABA and in inducing O_2^-^_ production at distinct ABA levels during leaf senescence. The enhanced expression levels of *OsNox2*, *OsNox5*, *OsNox6*, and *OsNox7* isoforms by ABA treatment exhibited different temporal patterns, and the transcript peaks of *OsNox2*, *OsNox5*, *OsNox6*, and *OsNox7* were at 3, 1, 6, and 12 h after incubating through a 100 μM ABA solution, respectively (Fig 8E–8H). In maize, a similar phenomenon has been observed in the expression levels of various *Zmrboh* isoforms, which exhibit distinct biphasic responding expression to ABA treatment; as a consequence, ROS continuously accumulate in the tissues of maize [16]. The diversity of the temporal transcriptions of *OsNox2*, *OsNox5*, *OsNox6*, and *OsNox7* in leaf tissues probably plays a complementary role in detecting ABA accumulation and inducing O_2^-^_ production.

However, a contradiction was detected between the decreasing transcript levels of *OsNox2*, *OsNox5*, *OsNox6*, and *OsNox7* isoforms from day 7 post-anthesis and the continuous accumulation of O_2^-^_ in senescing leaves in the leaf senescence stage. One possible explanation for this disparity is the presence of NADPH oxidases and other sources that generate ROS in senescing leaf cells. Numerous enzymes, including cell wall peroxidase, polyamine oxidase, oxalate oxidases, glycolate oxidases, and xanthine oxidases, and reactions, such as fatty acid oxidation, induce ROS generation [46, 47]. The same phenomena have been observed in rice leaves exposed to drought stress and maize leaves treated with exogenous ABA solution [9, 47]. Another interesting disparity was discovered on the transcripts of *OsNox6* and *OsNox7* in the leaves of the wild-type cultivar; the transcripts gradually increased on day 7 post-anthesis (Fig 6C and 6D). By contrast, the O_2^-^_ production rate of the corresponding blade remained relatively constant in the wild-type rice (Fig 1). These phenomena may result from the relatively high SOD activity in the wild-type leaves (data not shown) to eliminate O_2^-^_ accumulation by dismutation timely. Thus, the transcripts of *OsNox2*, *OsNox5*, *OsNox6*, and *OsNox7* in the *esl* mutant are probably involved in ROS generation in the initial stage of leaf senescence. Once leaf senescence started, the transcripts of the four *OsNox* isoforms were repressed because of Ca^2+^ deficiency in the senescing leaves of the *esl* mutant. Other oxidases in deteriorating cells subsequently induced ROS generation and accumulation.

## Supporting information

S1 Table O_2^-^_ production rate and ABA contents in two genotypes. (XLSX)

S2 Table O_2^-^_ production in detached leaf segments treated by exogenous ABA solution. (XLSX)

S3 Table O_2^-^_ production in detached leaf segments treated by exogenous ABA solution and DPI solution, respectively. (XLSX)

S4 Table The relative expressions of *OsNox* isoforms in two genotypes. (XLSX)

S5 Table Temporal expressions of *OsNox* isoforms in two genotypes during the grain-filling stage. (XLSX)

S6 Table Transcriptional analyses of *OsNox* isoforms in the detached leaf segments of two genotypes treated by exogenous ABA solution. (XLSX)

## Abbreviations

ABA: abscisic acid
DPI: diphenyleneiodonium chloride
*esl*: early-senescence-leaf
H_2_O_2_: hydrogen peroxide
NADPH: nicotine adenine dinucleotide phosphate
NBT: nitroblue tetrazolium chloride
NOX: NADPH oxidase
O_2^-^_: superoxide radical
qRT-PCR: quantitative real-time reverse transcription PCR
ROS: reactive oxygen species
